# 573. Discontinuation of Cabotegravir for PrEP: Challenges and Opportunities

**DOI:** 10.1093/ofid/ofaf695.182

**Published:** 2026-01-11

**Authors:** Alexander Kaplan, Dylan M Baker, Meredith H Lora, Valeria D D Cantos

**Affiliations:** Department of Internal Medicine and Pediatrics; David Geffen School of Medicine at UCLA, Los Angeles, CA; Emory University School of Medicine, Atlanta, Georgia; Emory University School of Medicine, Atlanta, Georgia; Emory University School of Medicine, Atlanta, Georgia

## Abstract

**Background:**

Guidelines recommend that individuals stopping long-acting injectable Cabotegravir (CAB-LA) pre-exposure prophylaxis (PrEP) who remain at risk for HIV acquisition take oral PrEP and complete quarterly HIV testing for 1 year after last injection; but, adherence to these guidelines is unknown. We describe the characteristics of individuals who discontinue CAB-LA and their adherence to post-discontinuation guidelines at an urban PrEP program in Atlanta.

**Table 1.**
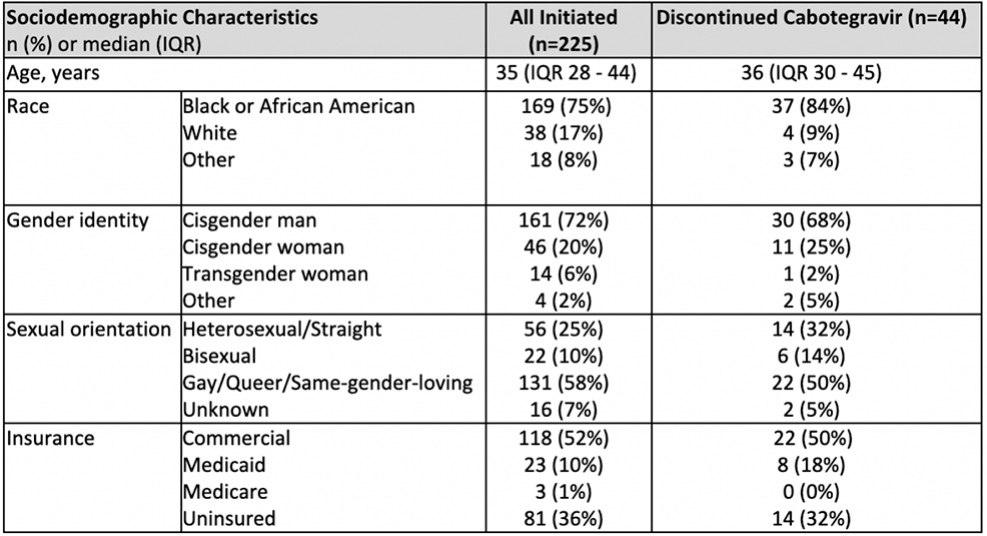
Patient Demographic and Clinical Characteristics

This table shows sociodemographic characteristics of all the patients that started CAB-LA and of those that discontinued CAB-LA within the PrEP clinic in urban Atlanta safety-net hospital.

**Figure:**
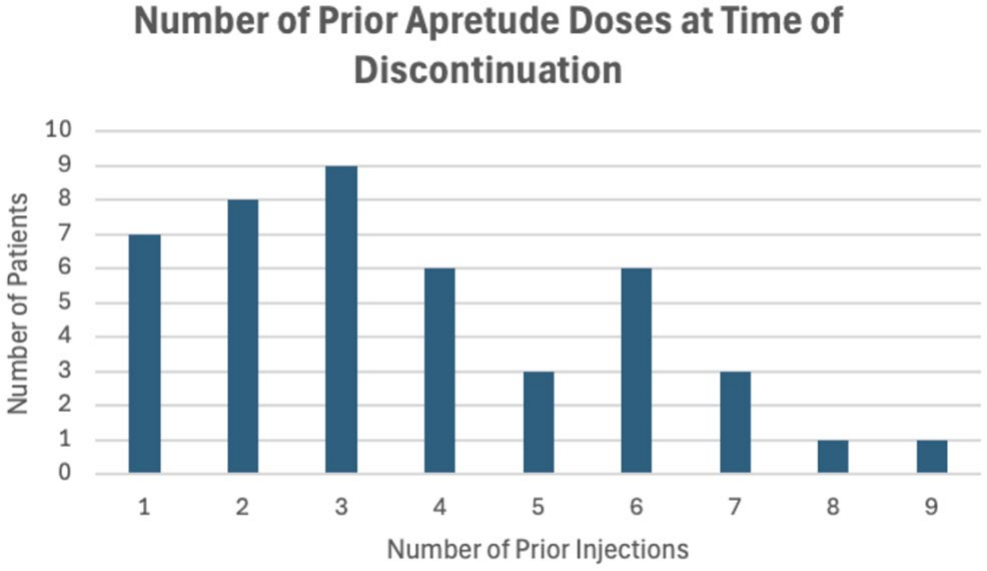

**Methods:**

Through retrospective chart review, we abstracted the sociodemographic and social determinants of health (SDOH) characteristics of individuals who discontinued CAB-LA from 12/1/2022 - 11/1/2024. We calculated the number of doses before discontinuation, reasons for discontinuation, proportion of individuals who transitioned to oral PrEP, and adherence to HIV testing schedule post-discontinuation.

**Figure:**
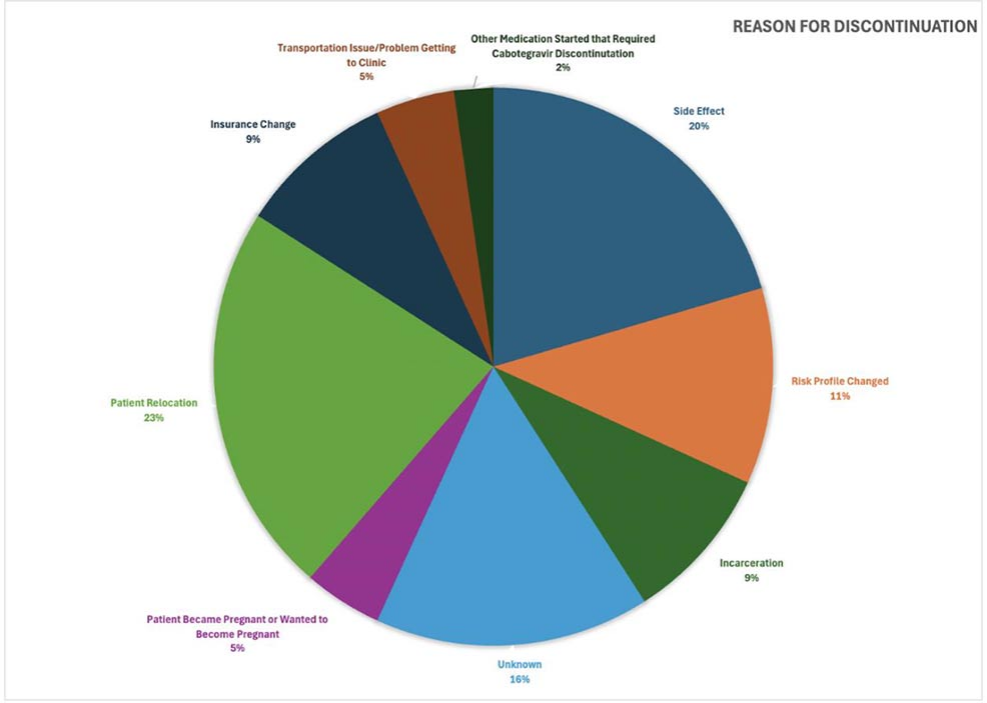

This chart shows the percentage breakdown for why patients in our clinic discontinued CAB-LA.

**Results:**

Of 225 individuals who initiated CAB-LA during the study period, 44 (19.6%) discontinued it, with 30 (68%) of all discontinuations occurring early after initiation (1 to 3 doses). The most common reasons for CAB-LA discontinuation were: 23% (10) patient relocation, 20% (9) side effects, 11% (5) risk profile change, 9% (4) insurance change, and 9% (4) incarceration. Common side effects included injection site reactions (4) and rash/urticaria (2). Of individuals who discontinued CAB-LA, 36% transitioned to oral PrEP. Of patients who did not transition to oral PrEP, 11% received subsequent HIV testing. There was one documented HIV seroconversion 3 months after incarceration related discontinuation.

**Conclusion:**

CAB-LA PrEP discontinuation in an urban PrEP program in Atlanta was similar to other PrEP programs in the US (Spinelli et al. 2024). Most individuals who discontinued CAB-LA neither transitioned to oral PrEP nor followed HIV testing recommendations post-discontinuation. With geographical relocation as the main reason for discontinuation, future interventions to develop a national CAB-LA PrEP clinic referral network are needed.

**Disclosures:**

All Authors: No reported disclosures

